# Trifocal femur fracture: Where should one begin? A case report and literature review

**DOI:** 10.1016/j.tcr.2026.101321

**Published:** 2026-02-24

**Authors:** Gianluca Cera, Giovanni Longo, Michele Dell'Orfano, Daniele De Meo, Francesco Maria Milella, Roberta Pica, Filippo Laurenti

**Affiliations:** a Orthopaedics and Traumatology Division, Department of Anatomical, Histological, Forensic Medicine and Orthopaedics Sciences, University “La Sapienza”, Piazzale Aldo Moro 5, 00185, Rome, Italy; b Ospedale di Faenza – Orthopaedics and Traumatology Department, Viale Stradone, 9, 48018, Faenza, Italy; c Azienda Ospedaliera San Camillo de Lellis – Orthopaedics and Traumatology Department, Viale J.F. Kennedy, 02100, Rieti, Italy

**Keywords:** Ipsilateral trifocal femur fracture, Intracapsular femoral neck fracture, Distal femoral intra-articular fracture, Antegrade intramedullary nail, Damage Control Orthopaedics (DCO), High-energy trauma

## Abstract

**Introduction:**

Ipsilateral, non-contiguous femoral fractures involving the femoral neck, shaft, and distal articular region are extremely rare and typically result from high-energy trauma. Their complex nature and rarity contribute to a lack of consensus regarding optimal surgical management.

**Case report:**

We report the case of a 72-year-old male involved in a high-energy motor vehicle accident who sustained a trifocal femoral fracture: an intracapsular femoral neck fracture (AO/OTA 31-B2, Pauwels type 3), a displaced diaphyseal fracture (AO/OTA 32-A2), and a complete intra-articular distal femoral fracture (AO/OTA 33-C2). Initial management included transtibial traction under a Damage Control Orthopaedics (DCO) protocol, followed by definitive fixation 10 days later using a single-stage stepwise surgical approach. Fixation consisted of an antegrade intramedullary nail (providing stabilization of the femoral neck and shaft fractures), 6.5-mm cannulated screws used exclusively for the intra-articular distal femoral fracture, and a LISS plate for the distal metaphyseal component.

**Results:**

Postoperative recovery was uneventful, with radiographic evidence of full fracture union and no signs of post-traumatic osteoarthritis at final follow-up. Clinical recovery was excellent.

**Discussion:**

This is one of the few reported cases in the literature involving a trifocal femoral fracture with this specific combination. Our surgical strategy allowed absolute stability of the distal articular fracture and relative stability for the shaft and femoral neck. A qualitative literature review revealed no standardized algorithm for implant selection or fixation sequence in such cases. Our experience supports a tailored approach based on fracture pattern, emphasizing the preservation of femoral head vascularity and anatomical reduction of joint surfaces.

**Conclusion:**

Trifocal ipsilateral femoral fractures require individualized surgical planning. The described combination of implants—used here for the first time—proved effective and may serve as a reference in similar complex trauma cases.

## Highlights

• Non-contiguous ipsilateral femoral fractures of proximal, diaphyseal, and distal segments follow high-energy trauma.
• In medial femoral neck fractures, especially in young patients, preserve the femoral head to prevent avascular necrosis.
• With simple lateral, diaphyseal, and complex distal (AO type C) femoral fractures, surgery should start distally and proceed proximally.
• Coronal malalignment, fracture malunion, and limb length discrepancy are the most common complications.
• Patients with this fracture pattern often require Damage Control Orthopedics, as Early Total Care is often not feasible.

## Introduction

Non-contiguous ipsilateral femoral fractures involving the proximal femur, diaphysis, and distal femur are typically associated with high-energy trauma and are poorly described in the current literature [1], [2].

The presence of both intracapsular femoral neck fractures and complete intra-articular distal fractures (AO/OTA type 33-C) [3] further complicates their management and is even more rarely reported [1], [4], [5].

Due to the rarity and complexity of these injuries, no consensus currently exists regarding their optimal surgical management [2], [5].

Although various traumatic mechanisms have been proposed, no agreement has been reached regarding the exact limb positioning at the time of injury that may lead to this fracture pattern.

This complex fracture pattern, often associated with high-velocity motor vehicle collisions or other high-energy mechanisms, poses a unique challenge to the orthopedic surgeon, as the choice of implant and the fixation sequence for each fracture segment are critical [2], [6].

Although some literature exists—primarily case reports and small case series—no clear algorithm has been proposed to define the timing, fixation sequence, and implant selection across all possible fracture pattern combinations [2], [5], [7].

It is crucial to note that fixation devices appropriate for isolated fractures may not be compatible in this complex clinical scenario [2], [7].

The aim of this study is to describe our clinical and radiographic experience and outcomes in the treatment of multifocal femoral fractures (via a single case report), and to provide a qualitative review of the relevant literature.

This case report presents a high-energy trifocal femoral fracture (intracapsular femoral neck, diaphyseal, and complete distal articular fractures) managed with an antegrade intramedullary nail, free cannulated screws, and a lateral LISS plate.

This combination of implants, not previously reported in the literature, enabled anatomical reduction and absolute stability of the periarticular distal fracture, along with functional reduction and relative stability for the diaphyseal and femoral neck fractures.

## Case report

A 72-year-old otherwise healthy male sustained a high-energy motor vehicle collision while driving.

On arrival at the emergency department, he presented with pelvic tenderness, a shortened and externally rotated left lower limb, a swollen knee, and intact distal pulses.

Initial diagnostics revealed: a left-sided basal pneumothorax with bilateral pleural effusion, fractures of the left 5th–8th ribs, xiphoid process fracture, nasal bone fractures, and a trifocal fracture of the left femur. He was therefore admitted to the trauma bay of our institution.

**Fig. 1:**
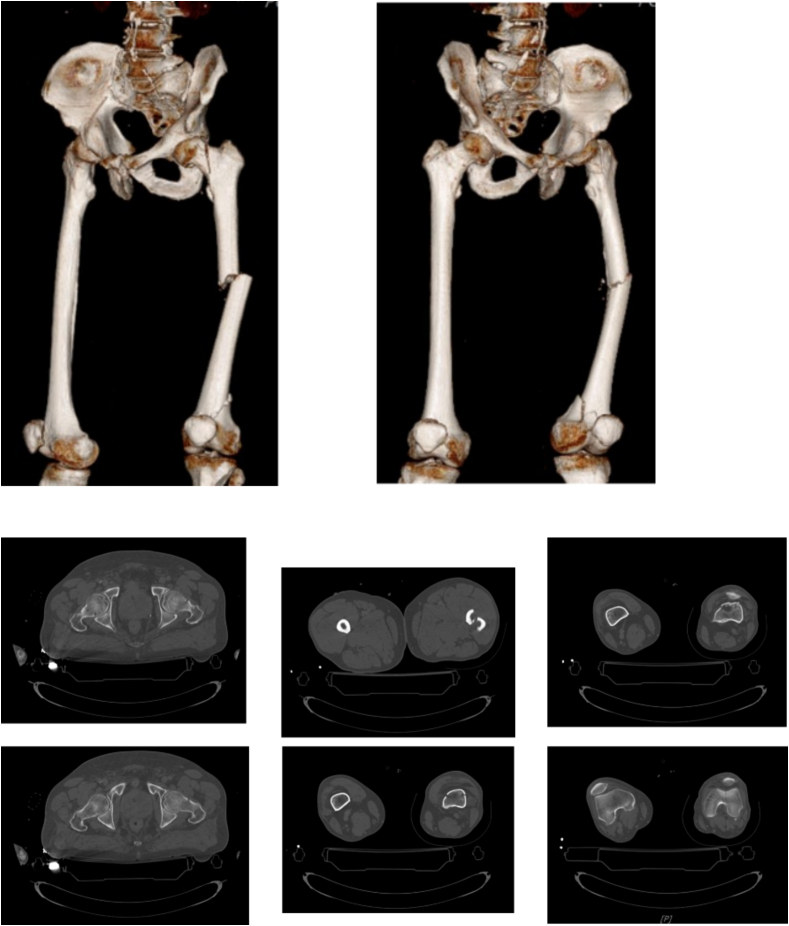
CT at the entrance to the emergency room.

The trifocal femoral fracture (Fig. 1) was classified as a transcervical obliquely oriented femoral neck fracture (AO/OTA 31-B2, Pauwels type 3), a displaced diaphyseal fracture (AO/OTA 32-A2), and a supracondylar fracture with intra-articular extension (AO/OTA 32-C2) [8].

No open fractures were present.

Due to the patient's clinical condition, a Damage Control Orthopaedics (DCO) approach was initially taken, with transtibial skeletal traction applied, and the patient was transferred to the intensive care unit (ICU) as he was not fit for early total care (ETC). Associated injuries included a pulmonary contusion complicated by bacterial superinfection and a temporal-basal cranial hematoma.

**Fig. 2:**
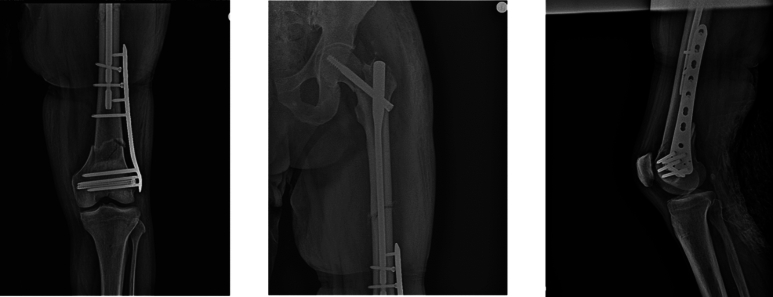
Immediate post-operative control X-ray.

Ten days later, once the pulmonary condition had stabilized, definitive surgery was performed in a single staged procedure with the following steps (Fig. 2):

1. K-wires to temporarily stabilize the femoral head and prevent displacement
2. 6.5 mm cannulated screw (Synthes) placed away from the future plate position to reduce the distal intra-articular fracture
3. Antegrade intramedullary nail with distal static locking
4. 11-hole 4.5 mm LISS plate (Synthes)

No cannulated screws were used for the femoral neck fracture.

The postoperative course was uneventful. The patient was advised to remain non-weight-bearing on the operated limb for the first three weeks, and to begin passive knee flexion-extension exercises within a controlled range during the first 15 days, followed by active exercises.

**Fig. 3:**
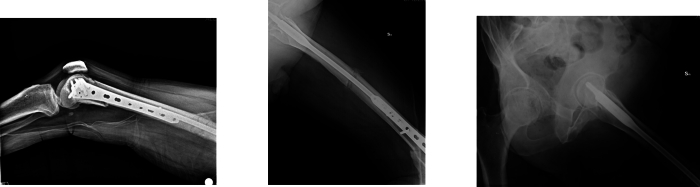
Control X-ray one month after surgery.

**Fig. 4:**
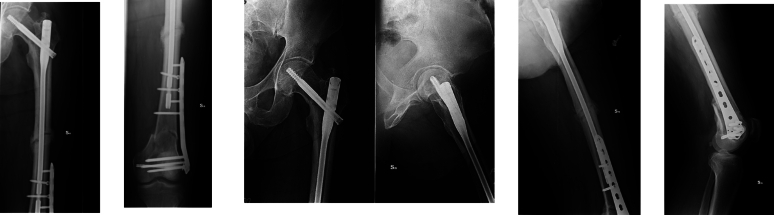
Control X-ray four months after surgery.

**Fig. 5:**
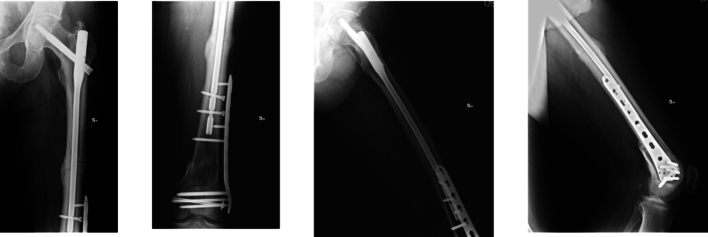
Control X-ray six months after surgery.

**Fig. 6:**
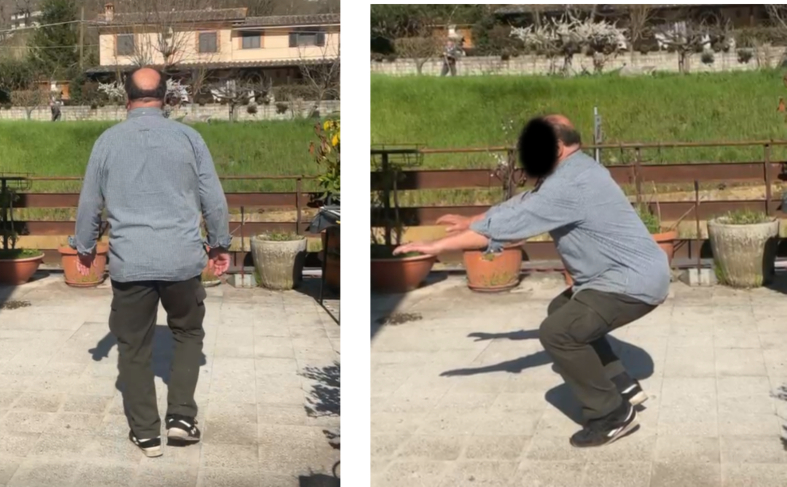
Clinical control at five year after surgery.

No radiographic signs of post-traumatic osteoarthritis were observed at final follow-up, and all fractures healed without complications, with excellent clinical recovery (Fig. 3, Fig. 4, Fig. 5, Fig. 6).

A qualitative literature review without date restrictions was performed using the MEDLINE (PubMed) and Google Scholar databases. Inclusion criteria were: studies reporting clinical and radiographic outcomes of patients treated for non-contiguous ipsilateral femoral fractures, English language, and publication in indexed journals. Exclusion criteria included lack of surgical technique description, absence of reported fixation sequence, lack of outcome data with validated scoring systems, and non-English or non-indexed articles.

The review identified 10 potentially relevant articles. Three were excluded due to non-English language, and two due to absence of fixation sequence or follow-up data, leaving five articles included in the review. A PRISMA flow diagram (Table 1) was created. These included three single-case reports and two small case series, encompassing a total of 16 patients with non-contiguous ipsilateral femoral fractures. Details of the included studies are presented in Table 2.

**Table 1:**
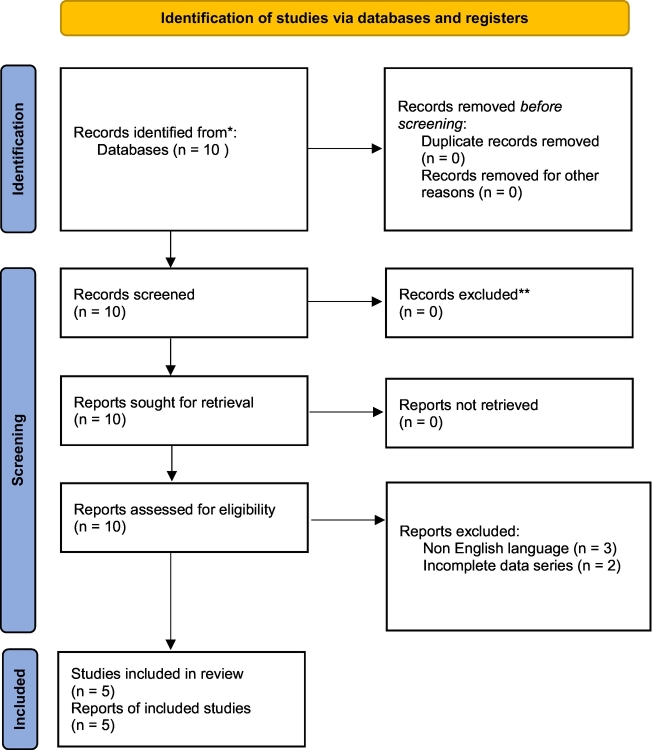
PRISMA flowchart for literature review.

**Table 2:** Literature review.

Study	Patients	Side	Trauma mechanism	Follow-up	Fracture pattern	Surgical treatment	Sequence of fixation	Complication	Reinterventions	Notes
Barei et al. [2]	1, F, 51 years	Right	High-energy vehicular accident	12 months	31A1.2 32A2b 33C1.1 34A1b	Long Gamma nail +95° condylar plate Rotula: dynamic tension banding	Proximal to distal Patellar fixation at 14 days after trauma	Distal femur malunion with valgus deformity Delayed healing of distal femur	Varus osteotomy of the distal femur at 16 months after trauma 1 cm limb length discrepancy	Early total care Delayed patellar fracture treatment
Griffin et al. [5]	7 5 M, 2F 19–63 years	4 right 3 left	High-energy vehicular accident	24 months	Pt 1: 31B2, 32A3, 33B3 Pt2: 31B2, 32A3, 33B1 Pt3: 31B2, 32A3, 33B3 Pt 4: 31B2, 32A3, 33B1 Pt5: 31B2, 32B3, 33B1 Pt6 31B2, 32A3, 33B3 Pt 7: 31B2, 32C3, 33B3	31: 4 cannulated screws 2 DHS 1 reconstruction nail 32: 4 retrograde nail 1 antegrade nail 1 reconstruction nail 1 plate 33: 4 screws 2 plate and screws 1 debridement Fixation device combination: Pt 1: femoral neck cannulated screws + femoral retrograde nail Pt 2: femoral neck cannulated screws + femoral retrograde nail + femoral condylar screws Pt 3: femoral neck cannulated screws + antegrade femoral nail, condylar screws Pt 4: femoral neck cannulated screws + antegrade femoral nail, condylar plate and screws Pt 5: DHS, femoral retrograde nailing, condylar screws Pt 6: DHS, femoral plate and condylar screws Pt 7: reconstruction nail + condylar plate and screws	Proximal to distal	1 femur varus deformity	None	5 pts. received definitive treatment within 24 h 2 hidden fractures of the femoral neck identified during the surgical procedure
Douša et al. [1]	5 (1 F, 4 M) Age 26–51 years	4 right 1 left	High-energy vehicular accident	2–13 years	31 A1 (*n* = 3) 31 B2 (*n* = 2) 32 A3 (*n* = 1) 32 B2 (*n* = 2) 32 C1 (*n* = 1) 32 C3 (*n* = 1) 33 B2 (*n* = 2) 33 C1 (*n* = 2) 33 C3 (*n* = 1) Open patellar fractures = 2	31: long femoral nail (Gamma, PFNA) in 3 patients 31: 2 dynamic hip screw plate-DHS (medial fracture) 32: 95° condylar plate (*n* = 2) 33: 95° condylar plate (*n* = 1) 33: cannulated screws (*n* = 2) Rotula: dynamic tension banding	3 pts.: proximal to distal 2 pts.: distal to proximal	Femoral shaft non-union (1/5) Femoral shaft malunion (1/5) Knee instability (1/5) Valgus femur deformity (1/5) Limb length discrepancy (1/5)	1 distal femur varus osteotomy 1 renailing 1 bone grafting	Associated lesions: 1 anterior cruciate ligament lesion 1 knee medial collateral ligament lesion Early total care
Meinberg et al. [3]	2 Male 41 years and 47 years	Right	High-energy vehicular accident	Pt 1: 24 months Pt 2: 30 months	Pt 1: 31-B2 32-B2 33-B3 Pt 2: 31-A2 32-B3 33-C1	Pt 1: twohole DHS + antirotational screw +16-hole stryker plate for distal femur Pt 2: three hole DHS + antirotational screw +13-hole stryker plate for distal femur	Proximal to distal	Delayed femoral fracture healing (30-months evident callus)	None	Early total care (on day of the trauma)
Bartoníček et al. [4]	1 pt., M, 51 years	Right	High-energy vehicular accident	8 months	31-B3 32-B2 33 A2	Antegrade intramedullary femoral nail	Proximal to distal	None	None	Surgical procedure within 24 h of arrival Open reduction and fixation of the femoral neck fracture

## Discussion

Trifocal femoral fractures involving the proximal, diaphyseal, and distal regions of the femur are exceedingly rare. Fewer than ten cases of trifocal femoral fractures with an AO/OTA type C distal fracture have been reported in the literature [1], [4], [5], [9], [10], [11], and only two of these included an intracapsular femoral neck fracture [4], [11].

The case presented in our report is therefore among the few published examples of a trifocal femoral fracture comprising a femoral neck fracture, a diaphyseal fracture, and a type C distal femoral fracture.

This type of femoral injury typically follows a somewhat predictable fracture pattern: a proximal femoral fracture is frequently associated with a diaphyseal fracture and a distal metaphyseal fracture, with or without intra-articular extension, or alternatively with a unicondylar fracture (medial or lateral).

The complexity of each fracture varies significantly across studies, ranging from non-displaced to severely comminuted and displaced fractures [1], [2], [4], [5], [6].

According to the literature, the fracture pattern distribution is as follows [1], [2], [5], [6]: medial proximal femoral fractures are the most prevalent (11 patients, 68.7%), with AO type 31B2 being the most common subtype (10 patients, 90.9%), followed by transcervical AO type 31B3 fractures (1 patient, 9.1%). Lateral proximal femoral fractures (AO 31A) account for 31.2% of all cases (5 patients), with 31A1 comprising 80% of these, and subtrochanteric fractures (AO 31A3) the remaining 20% (1 patient).

This distribution can be explained by the trauma mechanism, with vertically oriented femoral neck fractures typically resulting from axial loading along the femoral shaft when the hip and knee are flexed. In contrast, more lateral proximal femoral fractures may result from a different mechanism (e.g., lateral impact) or hip positioning (slight abduction) at the time of injury.

Patellar fractures are reported in two studies, both describing them as open fractures, although no specific classification was provided [1], [4].

Due to the high-energy trauma underlying these fracture patterns, patients frequently present with associated thoracic or head injuries and hemodynamic instability [2], [5], often necessitating ICU admission. For this reason, Early Total Care (ETC) is generally discouraged, and a staged approach based on the “window of opportunity” principle is currently the standard of care [12], [13].

Initial emergency treatment involves external fixation (EF) of the femur or femorotibial EF using various configurations depending on the fracture pattern, or skeletal traction. A CT scan is subsequently performed to better characterize the fracture morphology and articular involvement (if present), allowing for accurate preoperative planning.

When treating medial femoral neck fractures, especially in young patients, every effort should be made to preserve the femoral head and prevent osteonecrosis due to vascular injury. Therefore, it is critical to treat these fractures as early as possible, ideally during the emergency phase, to minimize the risk of avascular necrosis [12], [14], [15].

For this reason, fixation should generally proceed from proximal to distal [2], [6].

In other cases, the fixation sequence depends primarily on the specific combination of proximal and distal fracture patterns present.

Lateral femoral neck fractures associated with diaphyseal and simple distal femoral fractures (AO type A or B) can be managed with a long antegrade intramedullary nail and supplementary distal plate and screws, or with cannulated screws alone, proceeding from proximal to distal.

We believe that isolated use of a long proximal nail for treating associated AO type A distal femoral fractures [3] is feasible only when the distal fragment is large enough to accept at least three distal interlocking screws and when a secure bony corridor is available to ensure sufficient purchase and resistance to axial and torsional forces.

If the distal fragment is too short (<5 cm), we recommend the use of a short LISS plate with monocortical angular-stability screws placed proximally around the nail, with or without cerclage wires.

Regarding cerclage, some authors have suggested that cerclage around diaphyseal segments may damage the periosteum and potentially impair fracture healing.

In cases involving unicondylar type B fractures, 6.5 mm or 7.5 mm cannulated screws in combination with a 3.5 mm neutralization LCP plate may be used. Using screws alone may limit early mobilization due to reduced resistance to vertical displacement forces.

When femoral neck fractures are treated with cannulated screws, retrograde nails may still be used [2], [6] for associated diaphyseal fractures combined with simple extra-articular distal femoral fractures (type A), or a plate-and-screw construct may be applied for complex diaphyseal and distal femoral fractures (type B or C).

To our knowledge, no studies have reported the use of hip arthroplasty to treat displaced intracapsular femoral neck fractures in patients with this type of injury. This may be because the traumatic energy is typically dissipated along the femur, resulting in non-displaced or minimally displaced intracapsular fractures amenable to internal fixation. Only one study [6] described open reduction of a completely displaced medial femoral neck fracture using the Smith-Petersen approach, with no complications reported.

In cases involving a simple lateral femoral fracture, diaphyseal fracture, and complex distal femoral fracture (AO type C) [3], the ideal fixation sequence should begin distally and proceed proximally.

Dedicated 3.5 mm multi-hole plates and free cannulated screws can be used distally. When a multifragmentary metaphyseal fracture is present, an additional pre-contoured medial plate with autologous bone grafting should be employed; a lateral plate alone is insufficient to counteract medial cortical forces.

A stable lateral proximal femoral fracture can be treated with a DHS device. In cases of complex lateral or subtrochanteric fractures, an intramedullary device is recommended. The nail length should extend beyond the diaphyseal fracture and distal plate(s) used for internal fixation. This can be achieved with fixation extensions anchored to the plate.

The overall complication rate for surgical management of such fractures reported in the literature is 38%, with a reoperation rate of 19%. As expected, due to the considerable complexity of these injuries, coronal plane malalignment and limb length discrepancies are among the most common complications [1], [2].

Surprisingly, none of the studies reported the application of Damage Control Orthopaedics (DCO) which contrasts with today's recommendations [13].

Bartoníček et al. [4], Griffin et al. [5], and Kim et al. [6] all performed definitive surgical procedures on the day of trauma despite the presence of potentially life-threatening associated injuries (e.g., severe head trauma, rhabdomyolysis with acute renal failure, and pulmonary contusion).

Barei et al. [2] reported performing definitive surgery within 24 h of trauma in five of seven patients; no data were provided regarding the timing of surgery in the remaining two patients. In their cohort, two patients had severe abdominal injuries, two had significant thoracic trauma, and three had closed head injuries. Douša et al. [1] did not report any timing data.

According to current clinical practice, most of these procedures would likely be delayed due to the severity of associated life-threatening injuries.

In our case, we used an antegrade recon nail for fixation of the femoral neck and shaft fractures, cannulated screws for the intra-articular distal femur fracture, and a LISS plate for the supracondylar component.

This combination allowed prioritization of direct reduction of articular fractures while also achieving optimal stabilization of the femoral shaft via intramedullary nailing.

However, it is important to note that using a single implant for both the femoral neck and shaft fractures can present a drawback: should either fracture fail to heal, revision of the shared implant would jeopardize the stability of the other fracture site.

Additionally, the intracapsular fracture in our case—fixed solely via the cephalic screw of the intramedullary nail—was at risk for rotational displacement. Due to the Pauwels type III angulation of the fracture [8], placement of an anti-rotational screw was not feasible, as its threading would not extend beyond the fracture line.

Previous dogma and reports have often emphasized fixation of the proximal and distal segments, sometimes at the expense of the diaphysis, under the assumption that nonunion or malunion of the diaphysis is more manageable than complications involving either terminal segment.

While this may hold true from the surgeon's perspective, patients may still experience significant morbidity from this approach.

With modern implants, it is now possible to optimize the fixation of each component of these complex injuries, thus offering patients the best chance of an uncomplicated recovery.

## CRediT authorship contribution statement

**Gianluca Cera:** Supervision, Project administration, Investigation. **Giovanni Longo:** Writing – review & editing, Writing – original draft, Visualization, Validation, Software, Resources, Project administration, Methodology, Investigation, Funding acquisition, Formal analysis, Data curation, Conceptualization. **Michele Dell'Orfano:** Investigation. **Daniele De Meo:** Data curation. **Francesco Maria Milella:** Methodology. **Roberta Pica:** Formal analysis. **Filippo Laurenti:** Supervision.

## Informed consent

All the patients gave their approval via informed consent to publish their clinical and laboratory data, within utterly lawful respect of privacy.

## Ethical approval

Not required.

## Disclosure

The authors have no financial interest to declare in relation to the content of this article.

## Funding

No grant has been received for this study.

## Declaration of competing interest

None.
